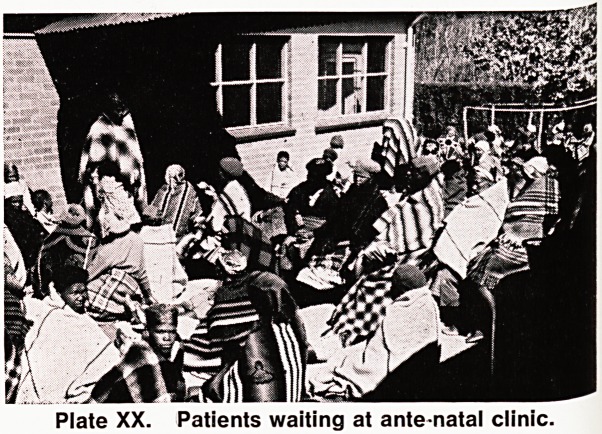# Government Medical Officer in Lesotho

**Published:** 1970-07

**Authors:** William E. Benney


### Government Medical Officer in Lesotho

William E. Benney, B.Sc., M.B., Ch.B., D.(Obst.) R.C.O.G.

Many people asked me why I was going abroad when apparently settled comfortably and securely in England, and I hastened to assure them that it was not on account of disillusionment with medical practice there! I had three equally-weighted reasons — to experience a temporary change from English general practice, to see something more of the world and its living and working conditions and finally, I hoped, to do some useful work in a place where doctors were scarce. I therefore approached the Ministry of Overseas Development in London, who arranged my appointment. I wished to work in Africa, preferably in a mountainous country, giving Uganda as first choice, partly influenced by Bristol's medical connections with Kampala, and Lesotho as second. At the time there were no vacancies in Uganda, and so arrangements were made for me to come to Lesotho.

### THE COUNTRY

I found that relatively few people knew where Lesotho (pronounced Lesootoo) was, but mention of Basutoland brought immediate recognition. Doubtless the country will be much more widely known in England now, as a result of the political unrest obtaining here in the weeks following the January, 1970, general election, the first since independence was granted in October, 1966. At that time the name of the country was changed from Basutoland to Lesotho. Lesotho is an enclosure within the Republic of South Africa, bounded by the Orange Free State, Natal, and Cape Province. Its size is about the same as Belgium, and the population about one million. The mighty Drakensberg range of mountains, reaching to a height of over 11,000 feet, arise in the north-east, and more central are the Maluti mountains, so that Lesotho is justly named the "roof of South Africa". The lowlands, with an altitude of 5,000 feet, occupy a strip along the north-west and south-west borders. The rains fall in the summer months, October to March, but have been very poor in recent years. By and large the climate is dusty and dry, as is the soil. When it does rain it is often very heavy, washing away the powdery top-soil and adding to the ever-increasing problem of soil erosion in the country. The towns, or "camps" are for the most part situated in the lowlands: there are nine. The capital is Maseru, with a population of 11,000.

Practically all the country's medicine is carried out in the hospitals. There are a few private general practitioners, who give good service, but of course on an economic basis, and the minority of people who can afford it will often prefer to attend them. These doctors have absolutely no access to hospital facilities. The

Plate XIV. Patients registering at Leribe dispensary.

main government hospital, Queen Elizabeth II Hospital, is in Maseru. It has 350 beds, and comprises separate male and female medical and surgical wards, children's ward (60 beds), gynaecology, maternity, male and female surgical extension (mainly long-term orthopaedics), male and female tuberculosis wards, a private wing which occupies the front of the hospital, a small laboratory, a mortuary, a theatre suite and the dispensary. There is an excellent new radio-diagnostic department, provided by West Germany and one of the mine recruiting organisations, and headed by a London-trained South African radiologist, who is the only South African doctor in government service. The other doctors are of assorted nationality—African, English, American, Indian, Korean, Phillipine etc. The dental surgeon — the only one in the country — is South African. There is a qualified anaesthetist who comes from Ireland. We have fairly recently been fortunate in acquiring a surgeon — a Pakistani from Manchester. To give us much-needed help we are blessed, in normal times, with two — or three — weekly visits by teams of South African surgeons, sponsored by a leading industrialist. These are mainly gynaecologists and orthopaedic surgeons, there being the greatest need for these specialities. They come from all parts of the Republic, from Friday to Sunday, giving their services free, and do "cold" surgery which has been collected from the various hospitals beforehand. A team consists of 2 consultants, 2 registrars, 2 theatre sisters and 1 anaesthetist, so that both theatres are kept busy.

In each of the camps there is a district hospital of 50 to 100 beds, run by one or two doctors according to size or, more often, availability of doctors! Outside the camps there are a number of mission hospitals—

French, Roman Catholic, Anglican and Seventh Day Adventist. These tend to be more stable as regards doctors and staff than do the government hospitals, and to be much better equipped. Some of the Europeans running them are Basutoland born and bred, and so have the advantage of an intimate knowledge of the life, language and customs of the Basuto, qualities not possessed by people like myself who come "out of the blue", as it were, for two years. Having been accustomed from the start of their careers to all branches of medicine, they are very versatile. One Swiss woman missionary doctor has been working up in the hills for 33 years and is still active. Such devotion, in very arduous conditions, makes one feel very humble.

**Plate XV. En route to Ramapepe clinic.**

I arrived in Lesotho on 1st October, 1968, and spent my first two months in Maseru, where I was introduced to the pattern of medicine in the country. Apart from emergencies which are brought in via casualty, all patients are seen in dispensary, a sort of combined G.P. surgery/hospital out-patient arrangement. Each patient pays 20 cents (2/- on the old exchange rate) and receives a small ticket 4in. x 3in. This serves as his prescription form and indeed is the only record there is — no outpatient notes are kept. If, as often happens on a future attendance, he has left his former ticket at home, or has lost it, there is no information as to what has happened before, which can be very frustrating. In the main hospital three or four consulting rooms are in operation at once, and there is no guarantee that a patient will see the same doctor two visits running. If admitted, a patient pays 20 cents daily, unless he be in a private room which is more expensive. Government employees have free out-patient but not in-patient treatment.

Whilst in "QE2", as the main hospital is commonly called, I learnt to do Caesarean section, which is relatively common, and much more so than forceps delivery, for contracted pelves, and separate inlet and outlet contraction, are of fairly frequent occurrence among the Bantu. Providing these are adequate there is seldom any trouble with uterine inertia, and I have not yet met a case with deep transverse arrest. I also learnt to do for the first time such minor operations as circumcision, and setting of fractures. After 11 years away from hospital work, apart from 6 months obstetrics, there was much to re-learn! I much regretted never having held an orthopaedic house-surgeon post — self-teaching from text books is not the ideal method of learning fracture work.

### LERIBE

After 2 months in Maseru I was moved to Mafeteng, the next camp 50 miles south, as second doctor to the hospital, the first being a Korean. I had just settled there after seven weeks when I had two days' notice to leave and move to my present hospital at Leribe (pronounced Lereebee), 60 miles north of Maseru. Unforeseen circumstances had suddenly arisen, leaving no doctor here. The notice was short owing to the breakdown of the telephone service, which in the camps operates 8 a.m. - 1 p.m. and 2 - 4.30 p.m., but not Saturday afternoon nor at all on Sundays. Eventually I was contacted via the police radio: I heard at 4.30 p.m. on Thursday and was packed and gone by noon Saturday.

Leribe is the largest of the districts, having a total population of about 60,000. The hospital was built in 1906, other parts having been added in the years since, so that it now accommodates about 100 patients. This figure is rather elastic, since when all beds are full patients sleep on the floor, and conversely, we are often not full. There are male and female wards, both having medical and surgical cases mixed, a children's ward with eight cots or beds — but with plenty of floor space for extra children and mothers — maternity ward with labour room, male and female tuberculosis wards with verandahs, operating theatre and a small fracture/minor ops. room. (Patients have to pay an extra 10/- if the main theatre is used.) The mortuary (no refrigeration) is behind the hospital. Corpses do not improve with keeping in the heat, and when the police request a post mortem, usually for murder, homicide or accident cases, I usually try to do it the same day or the next. Last year a two-ward mental block was built but the contractors went bankrupt before it was completed. Male mental patients are kept in a section of the prison, while females are accommodated in the general hospital ward, a procedure which often causes the place to be filled with their singing and shouting. But there are few complaints! Nearby the hospital is the dispensary, beside which is the outpatient department where injections and dressings are dealt with by a staff-nurse and attendants.

**Plate XVI. A clinic consultation.**

It is officially a two-doctor hospital, and has quite enough work for two, if not three, if there is to be any reasonable standard of medicine. For my first six weeks I was alone, and was then joined for four months by another English doctor who had worked for 18 months as a volunteer in a Catholic mission hospital and was then working temporarily for the government before leaving for Malawi. He left in July, 1969, since when I have been quite single-handed again. I had hoped to have some help from the return home of two Basuto doctors who trained in Israel and did their house-jobs in Nigeria and Zambia. They were due early this year but have not arrived, and it seems that they do not intend coming home, life being more attractive elsewhere. I feel it is very wrong that, in the extremely difficult circumstance of their homeland, they should not give two or three years service here, but apparently there is nothing to be done about it.

From the nursing aspect, the hospital is headed by an African matron or sister-in-charge, trained either at QE2 or in the Republic. Our matron has studied hospital administration in Australia, and is a very competent and pleasant person. We have a total of seven African staff-nurses, not all of whom, of course, are on duty at once. At night one staff-nurse is present in the hospital. Below them are men and women ward-attendants, rather like the orderlies at Frenchay in my far-off house-surgeon days, and, like them, some are outstandingly good.

### THE DAY'S WORK

The day starts about 8.30 a.m. with a round of the wards, usually fairly comprehensive on a Monday, but rather less so on other days, depending largely on how much work I see in front of me afterwards. At weekends I usually only see patients who are really ill or have been admitted during the night, but I make it a rule to go in every Saturday and Sunday morning. Any patients who will need operation with anaesthetic have a check of heart, lungs, blood pressure etc.; sometimes the staff-nurse, who of habit and necessity takes upon herself far more responsibility than does her counterpart in England, will already have starved a patient, and maybe given atropine premedication, expecting me to deal with him that morning. In such cases I invariably do so — it is only by working very much together that we can hope to keep things running at all. After the ward round is over, and one or two minor ops. done, then a cup of coffee in the office and a chat with matron over any matters needing attention or discussion. At last, and invariably with some reluctance, I make my way over to dispensary.

There are usually crowds of people at dispensary. They start arriving, Mondays to Fridays, from 7 a.m. onwards. They pay their 20 cents to register, and their names and villages are entered in a book. Registration is done by the dispensers, of whom we have three. They have three years training in Maseru, and their duties, apart from patient registering and dispensing of medicines, include assisting at post-mortems, administration of anaesthetics, and interpreting. I have found myself poor at learning the language — Sesotho — and have contented myself with a smattering of "terms of direction" — lie down, open your mouth, undress, and the like — so am completely reliant on an interpreter. The patients often come long distances and may have to catch a bus home, so I must try to finish dispensary by 4 p.m. or so. This involves working fast, much faster than my average 12 patients an hour in Bristol. My "record", though not one to boast about, was one day with a fairly large minor ops./fracture list in front of me when I saw 100 patients in 90 minutes. As can be imagined, the standard of diagnosis was not high! Unfortunately Lesotho does not have medical assistants, who have proved successful

Plate XVII. Kwashiorkor.

in other parts of Africa, and all patients want to "see the doctor", for however short a time. To my surprise, I found the proportion of trivial complaints much higher than I did in England: such things as "headache since last night", "a cold today", "rash which disappeared 2 days ago" and the like are very common.

At noon arrive recruits for the South African diamond and coal mines for their "medicals". These must of necessity be very brief — in any case they are all examined again and have chest x-rays on arrival at their destination, and I can only hope to pick up gross abnormalities which are fairly uncommon amongst mostly young men. I generally average half a minute on each — quick glance-over, heart, lungs, prodding of abdomen, and on to the next. The maximum number of people I have seen in a day so far is 300 — 200 patients and 100 miners. Working at that rate is very tiring, especially when it is very hot —

temperatures reach 95 degs. F. in the height of summer. Lunch break is 1 - 2.15 p.m. After dispensary has ended there are the various jobs in the hospital—operations, chest aspirations, fractures, difficult suturings (ward attendants do the simple ones), seeing ill patients I have sent in from dispensary without detailed appraisal, and such tasks. Routine work is usually finished by 5 or 6 p.m. Only emergency cases involve me working in the evening or night, which is just as well, as I find my energy much depleted by 6 p.m.

**Plate XVIII. Dispensing medicines from Landrover at clinic.**

### PATTERN OF DISEASE

The pattern of disease is that of a poor country which is not in the tropics — the only tropical-type diseases we see are occasional leprosy, and typhoid fever in the summer months. Typhoid seems to differ from text book characteristics in that pulse-rates are usually high — 120 to 150 — and I have not yet been able to palpate a spleen. People arrive when they have been ill for two or three weeks, sometimes moribund, but it is surprising how often they do recover. Treatment is by chloramphenicol, and diagnosis entirely clinical — after a bit of experience one can detect a case at sight. In my early days I used to ask the staff-nurse or dispenser "do you think this is typhoid?" — they were always right! We have no laboratory facilities in the hospital, and sending specimens by post to Maseru is so time-consuming and frustrating — often no specimen pots etc. — that I now rarely do so.

Pulmonary tuberculosis is rife and we have many cases. If ill they are admitted: if not, treated as out-patients. Drugs are streptomycin, INH and thiacetazone, with occasional P.A.S. Sputum examination is done at T.B. control in Maseru (based on WHO principles) and culture is carried out, but we have no drug sensitivity tests, so work quite in the dark in this respect. T.B. clinic is held once a month, but it is often difficult to persuade people to continue taking their tablets when they feel better, so relapse is common. I recently saw a man repatriated from the mines who had contracted pulmonary tuberculosis there and had three months treatment in the mine hospital, after which he was discharged and told to go to hospital at home. He looked very ill and I was surprised that he had been discharged in that state. After much discussion it transpired that he had been discharged **five years** ago and only just reported to us . . .

Assaults are very common, up to 20 a day on occasions. The Basuto are very ready for a fight, especially when fortified with locally-brewed millet beer. Weapons used are fists, feet, knives, spears, sticks, whips, stones — in fact anything readily to hand. Litigation invariably follows, the complainant coming to hospital with his "assault form" obtained from the police to be filled in by the long-suffering doctor. Often the injuries are very trivial, with not a single bruise to be seen, whilst others are serious — broken bones, haemo-pneumothorax, depressed skull fractures with extra-dural haemorrhage — and sometimes I do not see them until post mortem in the mortuary. Women are fond of assaults "with teeth", and in one recent case the victim had her left pinna neatly bitten off flush with her head! She was not unduly upset, but of course very keen to sue. For the more serious cases I have to attend court in due course. Notice is usually given in advance, but such is the difficulty in assembling all necessary people that the case is often postponed. One is then likely to be summoned suddenly by telephone on some future day, which can be quite disrupting to the hospital work but nevertheless must be complied with. Occasionally — usually for a murder case — I have had to travel the 60 miles to Maseru to attend High Court, and on some of these occasions was "not wanted" when I arrived — most annoying under the circumstances.

Venereal disease is a frequent reason for hospital attendance, and when very pushed I do not even inspect lesions, but take the patient's word for it. In general, it is not practicable to ask people to return for check-ups, except when it seems really important—there is just not time for such luxuries. I find I must accept a very much lower standard of medicine than in England — to attempt to maintain it at that level would be to court an early breakdown, in which case one would be of no use at all to the health service! Of course, I frequently have guilty feelings about this, but make myself realise that the situation must be accepted. Compromise or collapse!

Maize is the staple diet of the country — attempts to educate the people to grow wheat, vegetables, keep fowls and eat eggs etc., though progressing slowly, are yet far from having full impact. So malnutrition, pellagra and kwashiorkor are common, and we have many child patients covered with sores (treatment gentian violet and maybe penicillin), due partly to malnutrition, partly to lack of washing. In the remote villages water shortage is often a great problem — there were many deaths in one mountain region in 1969 due basically to water lack, and South Africa carried out an air lift to bring in food and water.

Gastro-enteritis is common, as is scabies. Appendicitis is uncommon and tetanus non-existent. The Basuto seem to suffer very much from constipation, and even babies a few days old are given enemas if their bowels fail to act often enough! No doubt this sets the pattern for later life. Burns and scalds are common in children, and soot is one of the commonly used "first-aid" measures before they are brought to hospital! Today a child was brought with sores of the scalp already treated topically with blue ink, but since I am as yet ignorant of any bactericidal properties of ink, perhaps it is early to criticise this!

Heart disease is uncommon except in the elderly, though one does occasionally meet cases of congenital and rheumatic heart disease. Surprisingly, there seem to be few cases of the late effects of syphilis. Many patients come to hospital very late, sometimes on account of long distances and difficult transport, so that we get fractures, dislocations, paraphimoses etc. which may be several days old before arrival, making effective treatment all the more difficult and unlikely to succeed well.

**Plate XIX. Young female witch-doctor.**

Typhoid fever occurs in the summer months and at the time of writing (March) there are several outbreaks in the country. Last week I went out with the health inspectors to a village group 20 miles away, on really bone-shaking tracks. There had been many cases with several deaths, and the epidemic had been in full swing for 4 weeks before word reached us. The Basuto usually look upon such diseases as due to evil spirits, and the situation has to get well-nigh out of control before it is reported by the chief. Water from wells and springs is usually drunk unboiled, and when we saw a few of the cases and the disgusting state of the springs it was not difficult to put two and two together. Two days later we went again on a mass inoculation scheme, expecting 1,000 people: 210 turned up. Trying to advance preventive medicine is a slow and laborious business.

The services of witch-doctors are still much in demand in Lesotho and they are consulted by many, either for treatment of disease, or for "prophylactic skin incisions", often at great cost. Sometimes patients are removed from hospital, even when recovering, to be taken home to the witch doctor. Conversely, witch doctors sometimes come to dispensary themselves for treatment, and even to be admitted. When a patient consults a witch-doctor, the latter always "knows" what is wrong with him or her. Hence the patient attending dispensary often does not expect to be asked questions about his symptoms — the doctor should know them already, because he is a doctor. A history has to be dragged out of the patient, who may then say what he **thinks** the doctor wants him to say, which can be very misleading. They love injections, and in my early days in QE2 I had many chest x-rays done for supposed haemoptyses, with usually normal findings. On talking this over with colleagues, I was told that the Basuto would invent a complaint of coughing up blood, this being thought a fairly reliable way to ensure injection treatment!

All district hospitals have x-ray plants, the films being taken and processed by the dispensers, with variable results. For the past four months, however, our plant has been broken down, so that patients have to be saved up and sent once or twice a week to the next camp Butha Buthe, 19 miles of dirt and stone road away in the Land Rover, if that too is not broken down — at present it spends about a third of its time in workshops for repair! These delays can be very frustrating, especially for fractures, which on occasions I have treated by "feel and guesswork". It is also not possible to have a check x-ray immediately after reducing a fracture.

For surgery, the anaesthetic used is ether, administered by a dispenser. In minor operations and fractures the "rag-and-bottle" method is used, but for bigger surgery—Caesarean section, etc.—we have the excellent EMO (Epstein-Mackintosh-Oxford) machine, a small portable device which is temperature-compensated and delivers any proportion of ether up to 20%. One of my predecessors obtained, through subscriptions from his church in England, a small extra attachment—a halothane inducer, which smooths induction nicely. For "rag-and-bottle" work, attendants keep the patient on the table until the second stage is over. One night when no dispenser could be contacted, I anaesthetised the patient myself with the EMO, then handed over to an attendant while I scrubbed up and proceeded with the Caesarean. All went well, though such a combination of activities can be somewhat harrowing!

### OBSTETRICS

Antenatal clinic is held twice a week, Wednesday and Friday, and is mainly run by the staff-nurses. I see patients as near as can be calculated at 36 weeks. Estimation of dates can be difficult—women often come up worried because they are "11 or 12 months pregnant", and conversely may deliver a full-term baby, quite adamant that they are only 5 months or so: this is usually because the husband was away at the mines 9 months ago, but home 5 months ago, and he will not be pleased to return home to find a baby conceived in his absence. I never do inductions for supposed postmaturity—one can never be sure of the dates, and since we have no luxuries like pitocin, infection would almost inevitably ensue, since most women have vaginal discharge. Indeed, discharge is accepted as normal, and a patient recently came complaining that she had none! It is rare for the head to be engaged at 36 weeks, or even at term, in the primigravid Bantu, which fact makes pelvic assessment more difficult.

By no means all women come to hospital for delivery, even if they have been coming to clinic, often just for the dried milk hand-out. Some deliver at home, or try to. The old mothers and grandmothers make them push hard with every pain from the onset of labour, and only when they are obstructed are they eventually brought to hospital. The enormous oedematous labia have to be seen to be believed, and vesico-vaginal fistulae are not uncommon. I have done several craniotomies and extraction of babies dead in obstructed labour owing to outlet contraction, under pudental block. One woman who came in *in extremis* died 10 minutes after arrival. In the case of normal deliveries, the mother goes home the next day, carrying her baby on her back: I have never seen a case of prolapse.

When I left QE2 for Mafeteng, the final word of advice from the S.M.O. was "beware the multip, who walks into hospital saying labour has stopped — she may have a ruptured uterus". Within a month such a case occurred—she actually came by bus, and walked into hospital, saying her labour had stopped 12 hours before. Appearance, general condition, pulse, and blood pressure were all normal, but the baby was dead. The head was rather high, so I was not able to use forceps, and proceeded to laparotomy. There was some blood in the peritoneal cavity. The baby practically fell out of a large rent low down on the posterior wall of the uterus: as soon as I removed it and the placenta, haemorrhage really began. The sucker wouldn't work, the light was poor and the generator-man out, so we had no electric light. I sent over to my house for my torch, and with the help of that was able to mend the rupture, tie her tubes, and close up, putting up a saline drip afterwards. The next day I managed to get two pints of blood from Maseru—there is a small blood transfusion service in the hospital there. After 10 days she went home well and happy.

I was recently faced with a difficulty new to me in manual removal of placenta. A small woman, a primigravida, had delivered tiny premature twins at home and then gone to a doctor-less mission hospital with retained placenta and much bleeding. She was sent on to me—30 awful stony-track miles in a truck, "bled out", pulse 150, B.P. 0. We had some plasma which I put up, and when her condition was a little better she was anaesthetised. Then the problem arose—the pelvic outlet was so small I could not get my hand in! In fact, I finally managed it, and with great difficulty removed the placenta from the uterus. The text-books do not tell one how to remove a stubbornly retained placenta if the uterus is inaccessible vaginally!

### DIFFICULTIES

When there are two doctors here, two outlying clinics, each about 15 miles distant, are visited weekly. Travel there is in the hospital Land Rover, along exciting rough tracks and through rivers. One dispenser comes. From 20 to 70 patients attend, according as to whether the doctor was expected. Really ill patients are brought back with us. Since I have been single-handed, it has not been possible to continue these clinics—patients have somehow to make their own way to us here.

Occasionally one has to visit the next camp for an emergency if the doctor is absent for any reason. One evening I had to go for an obstructed labour which ended with a Caesarean for a dead baby. The Land Rover was out of action, so I went in my own car. Whilst I was there it began raining heavily, and half way home I stuck in the road, now a quagmire. After $1\frac{1}{2}$ hours the rain had eased, and at last I was able to get mobile again, reaching home at 3.30 a.m. The next morning revealed a punctured flat tyre! One slowly learns to be philosophical over such happenings.

We are often short of drugs—at present we have no streptomycin or Mist:Mag. Trisil, and chloramphenicol is short. Often the trouble is administrative—getting the supplies from central medical stores in Maseru. Occasionally I have bought essential drugs like chloramphenicol and paraldehyde myself rather than be quite without them. In my early days when I was grumbling about drug shortage, an Edinburgh-trained Basutho doctor said "wait until you have to treat pellagra with aspirin, as I have had to!" Well, I have not yet had to do this, but I have treated syphilis with Ung-Calamine, telling the patients to come back in two weeks when I hoped we would have some penicillin again. One

**Plate XX. Patients waiting at ante-natal clinic.**

**must** give some visible treatment to patients—advice is never enough. And if one is to try and persuade more people to come for modern medical treatment rather than go to the witch doctors, it is necessary to pander to them more so than in England in order to gain their confidence.

What are my general impressions about being a doctor here in Lesotho? Although I did not expect life to be easy here, it is very much harder than I could have believed. I have had five real days off and away from the camp since a holiday last September. I miss contact and discussion with other doctors—and 'Med-Chi' meetings! In spite of seeing and dealing with so many people, I often feel I do not do as much good medicine in a day as I did at home, and there is virtually no "intellectual satisfaction" in the work. Against these facts, and very important, in the knowledge that one is the only doctor in the hospital, so that one's presence is essential, and that one is helping, albeit in a small and inefficient way, to keep modern medicine ticking over in a country which is slowly and painfully making its way towards fuller development. When my two years are up, I do not think I shall regret my stay here.

## Figures and Tables

**Plate XIV. f1:**
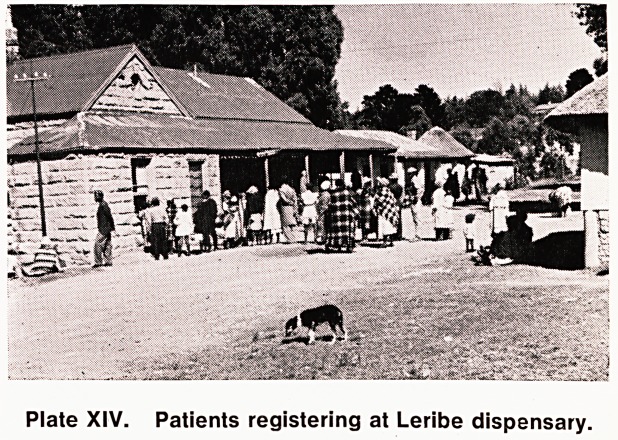


**Plate XV. f2:**
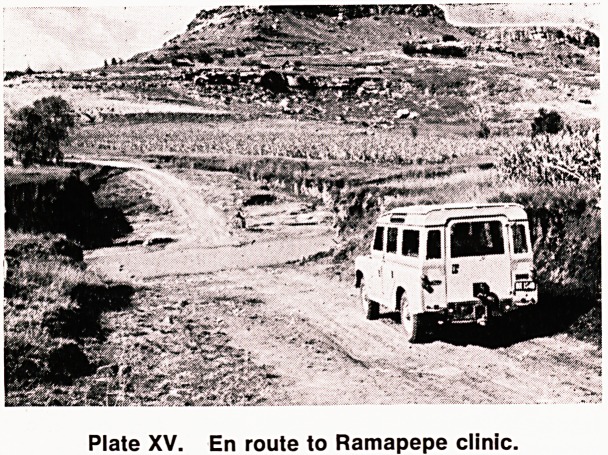


**Plate XVI. f3:**
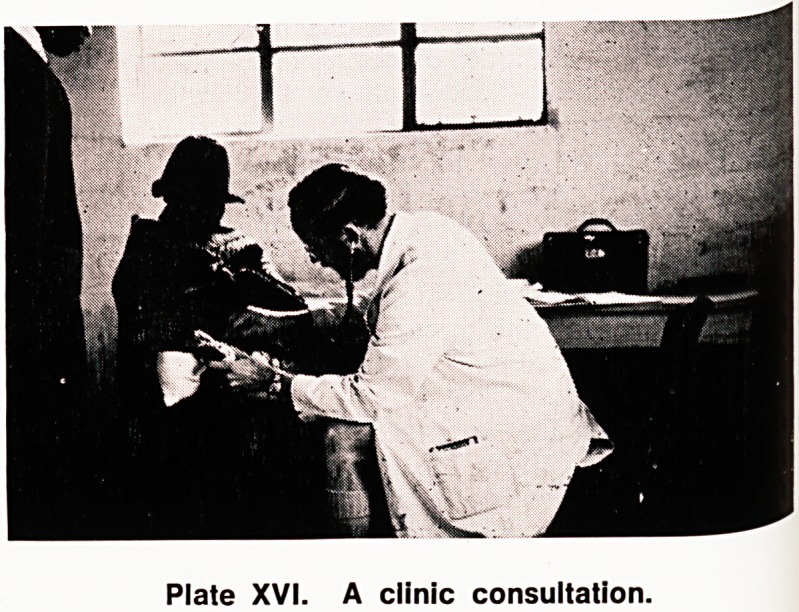


**Plate XVII. f4:**
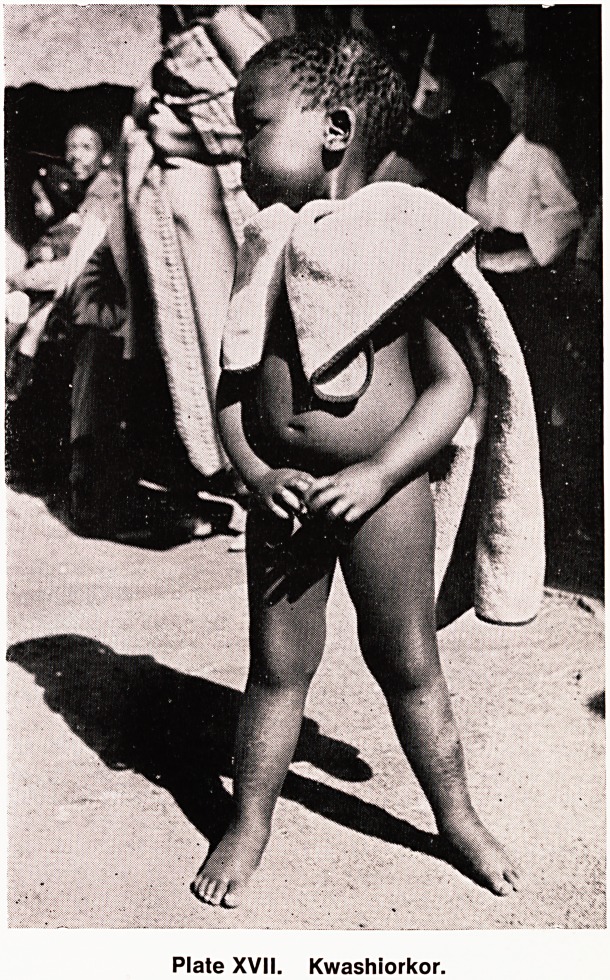


**Plate XVIII. f5:**
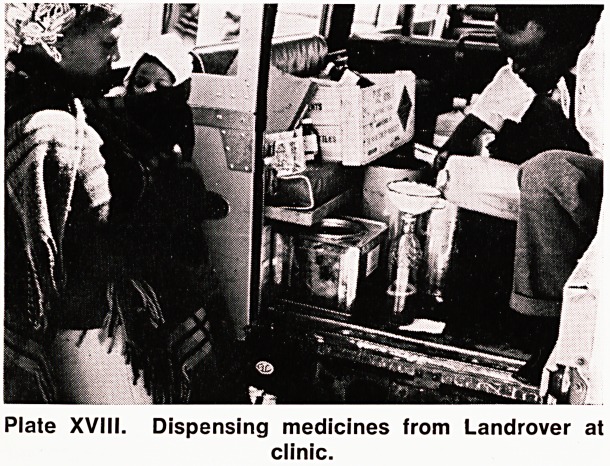


**Plate XIX. f6:**
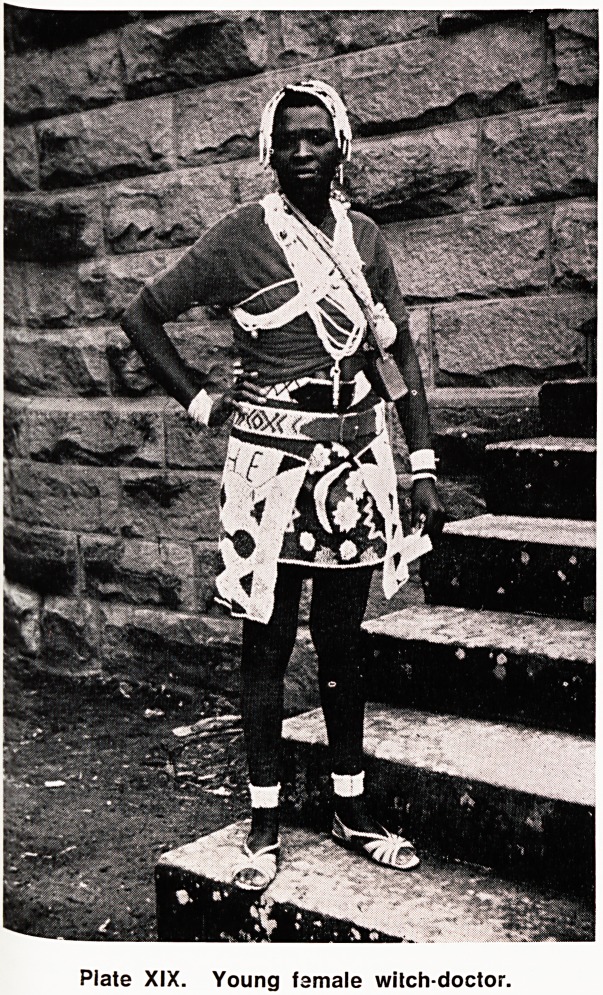


**Plate XX. f7:**